# Improved Anatomical Specificity of Non-invasive Neuro-stimulation by High Frequency (5 MHz) Ultrasound

**DOI:** 10.1038/srep24738

**Published:** 2016-04-20

**Authors:** Guo-Feng Li, Hui-Xia Zhao, Hui Zhou, Fei Yan, Jing-Yao Wang, Chang-Xi Xu, Cong-Zhi Wang, Li-Li Niu, Long Meng, Song Wu, Huai-Ling Zhang, Wei-Bao Qiu, Hai-Rong Zheng

**Affiliations:** 1 Institute of Biomedical and Health Engineering, Shenzhen Institutes of Advanced Technology, Chinese Academy of Sciences, Shenzhen, 518055, China; 2 School of Information Engineering, Guangdong Medical University, Dongguan, 523808, China; 3 Shenzhen Luohu People’s Hospital, Shenzhen, 518001, China

## Abstract

Low frequency ultrasound (<1 MHz) has been demonstrated to be a promising approach for non-invasive neuro-stimulation. However, the focal width is limited to be half centimeter scale. Minimizing the stimulation region with higher frequency ultrasound will provide a great opportunity to expand its application. This study first time examines the feasibility of using high frequency (5 MHz) ultrasound to achieve neuro-stimulation in brain, and verifies the anatomical specificity of neuro-stimulation *in vivo*. 1 MHz and 5 MHz ultrasound stimulation were evaluated in the same group of mice. Electromyography (EMG) collected from tail muscles together with the motion response videos were analyzed for evaluating the stimulation effects. Our results indicate that 5 MHz ultrasound can successfully achieve neuro-stimulation. The equivalent diameter (ED) of the stimulation region with 5 MHz ultrasound (0.29 ± 0.08 mm) is significantly smaller than that with 1 MHz (0.83 ± 0.11 mm). The response latency of 5 MHz ultrasound (45 ± 31 ms) is also shorter than that of 1 MHz ultrasound (208 ± 111 ms). Consequently, high frequency (5 MHz) ultrasound can successfully activate the brain circuits in mice. It provides a smaller stimulation region, which offers improved anatomical specificity for neuro-stimulation in a non-invasive manner.

Neuro-stimulation methods have been applied enormously in the neuroscience community due to their therapeutic utility for the treatment of neurologic and psychiatric diseases. They provide a powerful tool for the study of brain functions, for example investigating the mechanisms of cognition. At present, electrical-, optical-, magnetic-, and acoustic-mediated approaches have become the four main categories of methods used for neuro-stimulation^1^,^2^,^3^. Electrode-based electrical stimulation is the most widely accepted method for modulating the neural activities^4^. Deep brain stimulation (DBS) has an ability to stimulate deep brain areas by placing electrodes into the deep region of brain^5^. However it requires a neurosurgical procedure to conduct the stimulation which is a highly invasive method. Optogenetics has enabled the use of genetically encoded light to control the brain circuits^2^,^6^. It currently provides superior spatial resolution compared to all other brain stimulation methods. However, optogenetics does require the genetic modification, which is currently impossible for human beings. In addition, it is difficult to deliver light to the targeted neurons located in the deep brain.

At present, the most popular non-invasive brain stimulation methods are transcranial direct current stimulation (tDCS) and transcranial magnetic stimulation (TMS)^7^. tDCS achieves the neural stimulation by transmitting electrical current to activate target regions in the brain^8^,^9^. TMS utilizes magnetic energy to pass through the skull to modulate neural activities^10^,^11^. However the spatial resolution of both tDCS and TMS are noticeably worse than those invasive methods. They are difficult for specific activation of neuronal cells in a small region. In addition, they are not eligible for the stimulation of deep brain tissues.

Ultrasound has been used widely for diagnostic and therapeutic applications with its renowned reputation of being non-invasive, fine resolution and safe. Recent researches have demonstrated that ultrasound can successfully modulate the neural activity in brain slice cultures^12^, rodent animal^13^,^14^,^15^,^16^, monkey^17^, and even human being^18^,^19^,^20^. By transmitting acoustic waveforms into the brain, ultrasound is able to remotely and non-invasively excite neurons by activating voltage-gated sodium channels, as well as calcium channels^12^. The ultrasound mediated changes in neuronal activity are sufficient to trigger synaptic transmission in brain circuits. Most recently, the concept of sonogenetics has been proposed^21^. It has demonstrated that ultrasound can probably activate specific ultrasonically sensitized neurons in nematode. Therefore, ultrasound has provided a powerful tool for remotely modulating neural activity non-invasively.

Several ultrasonic parameters, including intensity, duration, pulse repetition frequency, duty cycle etc, influence the effect of neuro-stimulation^22^,^23^. Currently low frequency ultrasound (<1 MHz) is preferable due to its low ultrasonic attenuation when passing through the skull^3^. However, the stimulated area in the brain is hard to be specifically located, as the focal region of low frequency ultrasound is relatively large. It is a critical case, especially for the small animals such as mice due to their small brain size. According to the principle of acoustics, higher frequency ultrasound has the ability to generate smaller focal region^24^, which have a greater potential to ultimately reduce the stimulation area. For example, focused ultrasound transducer with 1 MHz frequency will generate a focal width of about 4.3 mm (Physical dimension of the transducer: 0.75 in. diameter, and 1.5 in. focal length, 1540 m/s speed of ultrasound). When using 5 MHz ultrasound, the width will decrease to about 0.86 mm. High frequency (5.7 MHz) ultrasound has been used to produce biological effects on nerve conduction blockage^25^. This study examines the feasibility and effectiveness of using high frequency, i.e. 5 MHz, focused ultrasound to perform the neuro-stimulation on mice, and verifies its anatomical specificity of neuro-stimulation *in vivo*. Our results indicate that 5 MHz ultrasound can efficiently penetrate through the mouse skull. It can stimulate brain to evoke electromyography and movement responses with improved anatomical specificity. The values of equivalent diameter (ED), which describes the size of effective stimulation region, and response latency are significantly smaller than those using 1 MHz ultrasound.

## Results

### Theoretical evaluation of stimulation region

**Figure 1:**
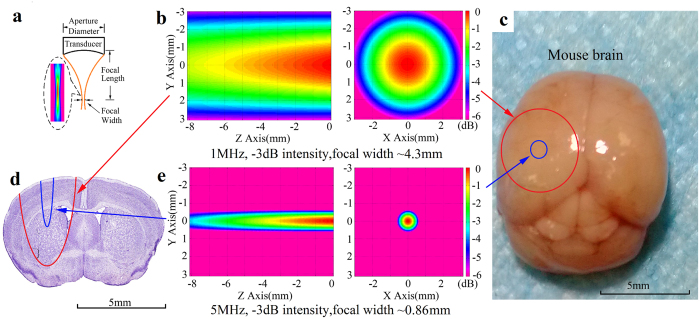
Theoretical comparison of the stimulation region using 1 MHz and 5 MHz ultrasound. (**a**) Schematic diagram of focused ultrasound transducer. (**b**) Simulation results of 1 MHz ultrasound intensity, Y-Z plane (left), Y-X plane (right). (**c**) A mouse brain with its left hemisphere marked by two circles. One is for 1 MHz, −3 dB focal width (red, outside circle), the other one is for 5 MHz, −3 dB focal width (blue, inside circle). (**d**) Coronal section of a mouse brain, left side marked by two semi-ellipses which present 1 MHz, −3 dB sonication region (red, outside) and 5 MHz, −3 dB sonication region (blue, inside). (**e**) Simulation results of 5 MHz ultrasound which is similar with (**b**).

Figure 1a illustrates the outline of a concave transducer with its ultrasonic beam. Most of the acoustic energy concentrates on a region named as focal region. The width of focal region is proportional to the acoustic wavelength and the f-number of the transducer^26^. In the application field of biomedical studies, increasing the center frequency of a transducer is an efficient method to reduce the focal width. In order to theoretically evaluate the stimulation region by different ultrasound frequencies (1 MHz and 5 MHz), simulation was carried out by the Field II program^27^. Spatial peak temporal average intensity (I_spta_), was used to represent the intensity of the ultrasound in this study. As the acoustic field simulation maps shown in Fig. 1b,e, the −3 dB intensity focal width of 1 MHz ultrasound is about 4.3 mm, while that parameter is significantly decreased to 0.86 mm for 5 MHz ultrasound. The Fig. 1c,d show the comparisons of focal width and depth for two transducers with 1 MHz and 5 MHz ultrasound frequency. Obviously, it tends to obtain a much smaller focal region using 5 MHz ultrasound than using 1 MHz ultrasound. So that 5 MHz ultrasound has the potential to achieve a much smaller stimulation region than 1 MHz ultrasound on the mouse brain.

### Acoustic measurement and compensation

**Figure 2:**
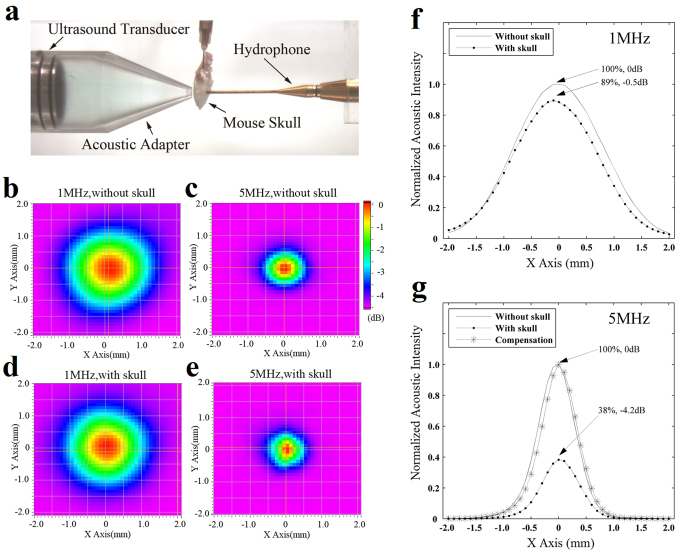
Acoustic intensity measurement and results. (**a**) Experimental setup for acoustic intensity measurement. (**b,c**) Are acoustic intensity distribution maps scanned without mouse skull by 1 MHz and 5 MHz ultrasound, respectively. (**d,e**) Were similar maps with mouse skull blocking. The intensities had been normalized to the peak value for each map. (**f**) Normalized intensity curves of 1 MHz from section planes of (**b,d**) at Y = 0 mm. (**g**) Normalized intensity curves of 5 MHz from section planes of (**c,e**). The dash line with asterisks marked shows the compensation result.

The acoustic intensity maps were measured by the 3D ultrasound intensity measurement system (Fig. 2a). By comparing the normalized distribution maps of 1 MHz and 5 MHz ultrasound without the influence of skull (Fig. 2b,c), and the similar maps with skull (Fig. 2d,e), it can be seen that there are no obvious changes in terms of the size and location of focal regions when using different ultrasound frequencies. For more precise comparisons, the measured data were plotted on curves. Figure 2f shows normalized intensity curves of 1 MHz by sectioning Fig. 2b,d at Y = 0 mm. The solid line and the dotted line describe the intensity distribution along X axis without and with skull, respectively. The dotted line has lower peak value (89%, −0.5 dB) compared with the solid line (100%, 0 dB). Figure 2g shows the normalized intensity curves of 5 MHz. The dotted line has a much lower peak value (38%, −4.2 dB) compared with the solid line (100%, 0 dB). Acoustic compensation was added by increasing the amplitude of the ultrasound waveform in the driving part. The dash line with asterisks in Fig. 2g shows the acoustic intensities after acoustic compensation, which has peak value similar with the solid line. The difference of acoustic intensity after passing the skull is very small (<5%) for 1 MHz and 5 MHz ultrasound after acoustic compensation, which enable a fair comparison between different methods.

### Motion response evoked by 5 MHz ultrasound

**Figure 3:**
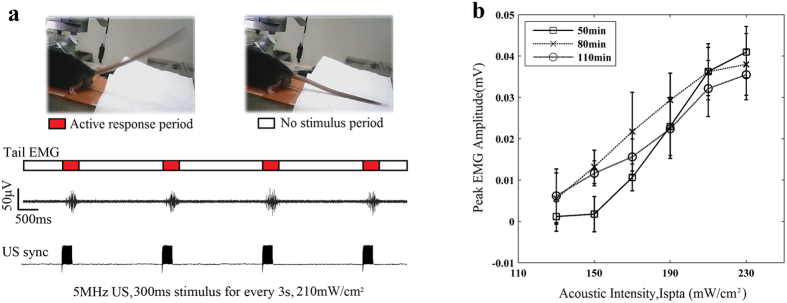
Illustration of motion responses evoked by 5 MHz ultrasound stimulus. (**a**) EMG signal (upper waveform) acquired from a mouse tail shows the motion responses evoked by ultrasound stimuli. The bottom waveform is a synchronous signal indicating the stimuli phases. The red bars represent some active response periods, while the white bars represent no stimuli periods. (**b**) Three curves (n = 20 stimuli) represent trends of the peak EMG amplitudes evoked by 5 MHz ultrasound varying with different acoustic intensities at three different time frames, i.e. 50 min, 80 min, and 110 min after the first anesthesia injection, respectively. The error bars show the standard deviations.

According to the experiments, 5 MHz ultrasound can successfully activate the brain circuits in mice to induce tail flicks. Figure 3a shows EMG signals from the tail evoked by 5 MHz ultrasound with I_spta_ = 210 mW/cm^2^, and the synchronous signals which were used for indicating the stimuli phases. Video can be found from Supplementary material (Supplementary Video S1). Figure 3b shows the peak EMG amplitudes varied with different acoustic intensities of 5 MHz ultrasound at three different time frames, i.e. 50 min, 80 min, and 110 min after the first anesthesia injection. These three curves have a similar pattern that is the peak EMG amplitude increases as the acoustic intensity goes up. The stimulation is stable and can be maintained in long time (>60 minutes) with high frequency ultrasound. All of the error bars stand for the standard deviations.

### Analysis of EMG signals evoked by 1 MHz and 5 MHz ultrasound

**Figure 4:**
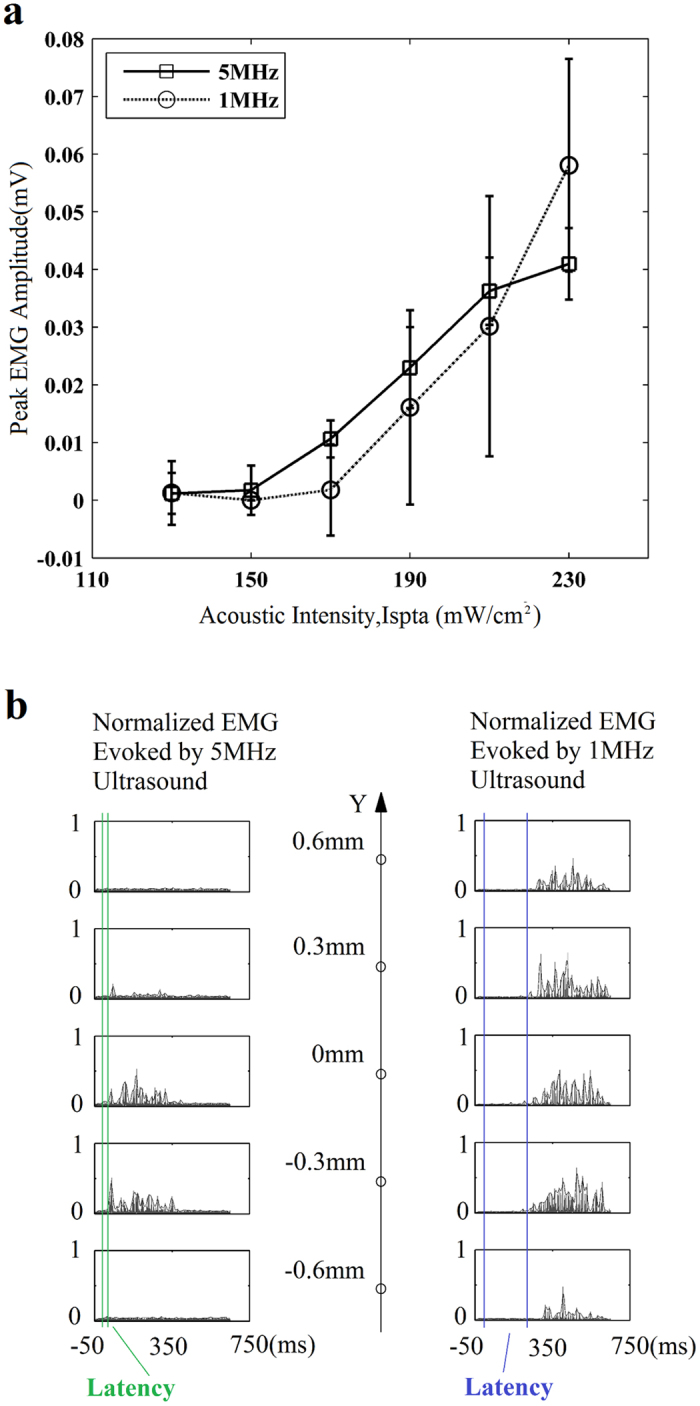
Comparisons of EMG amplitude and waveforms evoked by 5 MHz and 1 MHz ultrasound stimuli. (**a**) Peak EMG amplitudes (n = 20 stimuli) evoked by 5 MHz (solid line) and 1 MHz (dash line) ultrasound, with different values of I_spta_ ranging from 130 mW/cm^2^ to 230 mW/cm^2^. (**b**) Normalized EMG waveforms evoked by 5 MHz (on the left) and 1 MHz (on the right) ultrasound at 5 stimulation sites along Y axis. The duration time of each waveform is 800 ms, which includes 50 ms before the onset of each stimulus. The response latency between stimulus onset and EMG onset is indicated with two vertical lines.

Figure 4 shows the results of comparison experiments on the same group of animals with high frequency and traditional frequency ultrasound stimulation. The motion responses were characterized by EMG signals. Figure 4a illustrates that the peak EMG amplitude increases gradually when increasing the acoustic intensity with 1 MHz (dash line) and 5 MHz (solid line). The compensated acoustic intensities range from 130 mW/cm^2^ to 230 mW/cm^2^. The stimulation position was fixed at a reference point, which located around at 3.5 mm lateral to the midline and 7.5 mm posterior to the rear corner of mouse eyes, and was carefully modified until having the most sensitive motion response to ultrasound stimulation. These two stimulation methods using different ultrasound frequencies have a similar effect on inducing tail flicks to acquire EMG signals on live animals at this point.

However, the situation is changed when moving the stimulation point. Figure 4b plots the rectified and normalized EMG waveforms evoked by 5 MHz and 1 MHz ultrasound at five stimulus sites along Y axis, respectively. In the case of 5 MHz stimulation, only two points (i.e. Y = 0 mm, and Y = −0.3 mm) have obvious EMG waveform. Besides that, weak waveform can be found at the second point (Y = 0.3 mm), but almost no EMG signal was acquired at the farther points of Y = 0.6 mm and Y = −0.6 mm. For 1 MHz stimulation, the result was different. Strong EMG signals could be acquired at all points. In a word, the quantity of successfully evoked waveforms of 1 MHz ultrasound is larger than that evoked by 5 MHz. Moreover, the EMG response latency of 1 MHz ultrasound is greatly larger than that of 5 MHz. The following section will show more data of the latency.

### Quantitative comparison of anatomical specificity

**Figure 5:**
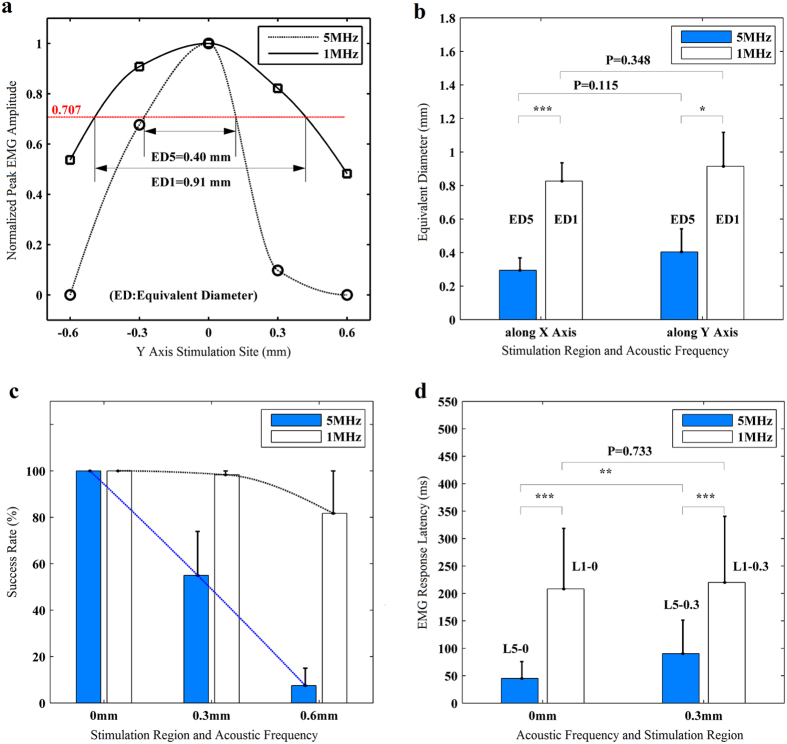
Comparisons of educed parameters from EMG evoked by 5 MHz and 1 MHz ultrasound stimuli. (**a**) Two fitted curves were formed by piecewise cubic interpolation from two groups of circle dots set and square dots set, respectively. Each of the circle dots represents the means (n = 6 stimuli) of peak EMG amplitudes evoked by 5 MHz ultrasound at a site, while that of the square dots by 1 MHz. (**b**) Comparison of the mean values (n = 5 mice) of ED5 and ED1 measured along X Axis and Y Axis. (**c**) Comparison of the success rate of the EMG signals (n = 5 mice) evoked by 5 MHz and 1 MHz ultrasound at the stimulation regions which are 0, 0.3, and 0.6 mm from the reference point, respectively. (**d**) Comparison of the mean latency of EMG response to 5 MHz and 1 MHz stimuli at the stimulation regions which are 0 and 0.3 mm from the reference point(L5-0 means latency of 5 MHz at 0 mm site, L1-0.3 means latency of 1 MHz at 0.3 mm site). All of the error bars stand for standard deviations.

We have made a quantitative comparison of the anatomical specificity of brain stimulated by 1 MHz and 5 MHz ultrasound. Figure 5a shows the stimulation profile of 1 MHz and 5 MHz ultrasound with equivalent diameter (ED) values (n = 6). The stimulation profiles are demonstrated by two fitted curves. One curve was fitted by the points calculated from the normalized peak EMG amplitude of 1 MHz ultrasound, the other curve was from that of 5 MHz. These curves were fitted by piecewise cubic interpolation. The horizontal axis value stands for the stimulation sites along Y axis. Obviously, the 5 MHz curve has sharper and narrower shape than that of 1 MHz. It can be measured that ED5 (ED5 stands for the ED value of 5 MHz ultrasound) value is 0.4 mm and ED1 (ED1 stands for the ED value of 1 MHz ultrasound) value is 0.91 mm. To investigate the total values of ED1 and ED5, statistic data of five mice are plotted on Fig. 5b. Along X axis, ED5 is 0.29 ± 0.08 mm, ED1 is 0.83 ± 0.11 mm, they are statistically different from each other (Paired-sample t-test, t = −11.854, 4 d.f., p < 0.001). Along Y axis, ED5 is 0.40 ± 0.14 mm, ED1 is 0.91 ± 0.21 mm, they are also statistically different from each other (Paired-sample t-test, t = −3.3424, 4 d.f., p < 0.05). It can be clearly concluded that the ED5 value is quite smaller than ED1. In other words, the anatomical specificity is improved by stimulated with 5 MHz ultrasound, as compared with 1 MHz.

### Evaluation of success rate and latency

Beside of ED values, the success rate of EMG signals evoked by 5 MHz and 1 MHz ultrasound at three stimulation sites are shown in Fig. 5c. The success rate of 5 MHz stimulation decreases from 100% to 7.5%, while that of 1 MHz stimulation varies from 100% to 82% as the stimulation site moves away from the reference point. Obviously, the success rate of 5 MHz ultrasound decreases much quickly in comparison with that of 1 MHz. This variation trend of success rate between 5 MHz and 1 MHz stimulation implies that 5 MHz ultrasound obtaining smaller anatomical region for brain stimulation.

The latency of the EMG responses to 5 MHz stimulation is significantly shorter than that to 1 MHz stimulation. The mean latencies of EMG response to 1 MHz and 5 MHz ultrasound at stimulation regions which are 0 and 0.3 mm from the reference point, are L5-0 = 45 ± 31 ms, L1-0 = 208 ± 111 ms, L5-0.3 = 90 ± 61 ms, L1-0.3 = 220 ± 121 ms, respectively (Fig. 5d). The statistically difference between 1 MHz and 5 MHz is distinct (Independent-sample t-test, t = −6.844, 26.564 d.f., p < 0.001). Moreover, the statistically difference of latency for 5 MHz ultrasound between these two stimulation regions is distinct too (t = −3.193, 26.564 d.f., p < 0.01). But statistically difference of latency for 1 MHz ultrasound between these two stimulus regions is indistinctive (t = −0.344, 45.639 d.f., p = 0.733).

### Evaluation of temperature and safety

The peak value of temperature elevation is 0.2 °C induced by 1 MHz ultrasound with 300 ms stimulus duration. The peak temperature elevation for 5 MHz ultrasound with the same duration is 1.6 °C, which is higher than the value induced by low frequency ultrasound. When the duration for 5 MHz ultrasound stimulus is decreased from 300 ms to 150 ms, the temperature elevation is decreased to 0.8 °C.

**Figure 6:**
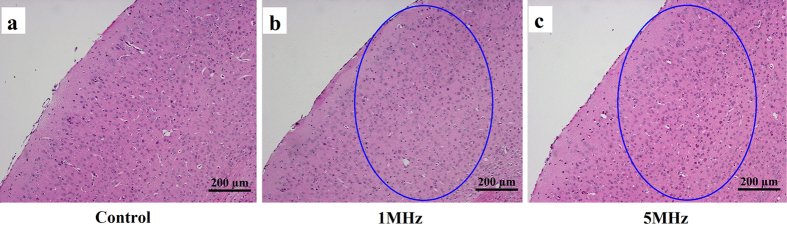
HE stain for brain slices. (**a**) HE stain for control mouse without performing ultrasound stimulation. (**b**) HE stain for mouse under brain stimulation by 1 MHz ultrasound for 30 min. (**c**) HE stain for mouse under brain stimulation by 5 MHz ultrasound for 30 min. The ellipses indicate the regions where ultrasound stimulation occurred.

Figure 6a–c show the results of HE stain. The elliptical circles represent the ultrasound stimulation region. 1 MHz and 5 MHz ultrasound did not induce obvious morphological changes in the stimuli regions, as compared with the control mice. In addition, there is no obvious tissue bleeding or cell necrosis in the tissue stimulated by ultrasound. These results demonstrate that 1 MHz and 5 MHz ultrasound are safe for the neuro-stimulation.

## Discussion

In contrast with other neuro-stimulation methods, ultrasound offers a number of advantages, such as non-invasive procedure, MRI compatibility, and synchronous monitoring, etc. This study investigated the feasibility of applying 5 MHz ultrasound to achieve neuro-stimulation on mice with improved anatomical specificity. This study examined the feasibility and effectiveness of using higher frequency (5 MHz) ultrasound to evoke motor responses of mice. The results show that 5 MHz ultrasound can efficiently pass through the mouse skull and stimulate brain to evoke motion potentials and movement responses. It provides a reduced focal region, which thus offers an improved anatomical specificity in neuro-stimulation in a non-invasive manner. It also helps to explore neural circuits which are sensitive to ultrasound stimulation.

The attenuation induced by the skull on ultrasound was one of the principal reasons to limit high frequency ultrasound for the brain stimulation. However, the situation is different in mouse studies. Although 5 MHz ultrasound suffers from 4.2 dB energy loss when passing through the skull, it can be compensated by adjusting the power of driving waveforms. The intensity of the acoustic power in the brain was kept at the same level with traditional method, which made sure 5 MHz ultrasound stimulation was safe for the animal. By applying an acoustic compensation procedure, 5 MHz ultrasound can effectively pass through the intact mouse skull, resulting in almost identical acoustic intensities in mouse brain as 1 MHz ultrasound. It is also possible to apply high frequency ultrasound to primate or even human to reduce the focal region by the aid of compensation procedures. Array based ultrasound technology and time reversal algorithm^28^,^29^,^30^ will be helpful to do the acoustic compensation.

According to the experimental results in this paper, 5 MHz ultrasound can be successfully applied to the brain stimulation on mice. From the EMG data and the real-time videos, 5 MHz ultrasound can achieve similar stimulation performance on mice compared with the published work. The stimulation effect is similar with traditional method when applying acoustic compensation. The focal region decreases about five times in contrast with the one in 1 MHz ultrasound stimulation theoretically. The activation region should be reduced accordingly, so that the anatomical specificity can be improved when stimulating the brain circuit. The acoustic adaptor shown in this paper was actually a container for the ultrasonic gel which allowed the ultrasound to pass to the brain reliably. The structure was designed to have little influence to the ultrasound beam. The acoustic measurement in Fig. 2 could demonstrate this.

By performing multi-sites stimulation to a certain region using 1 MHz and 5 MHz ultrasound, variations of the movement reaction were used to evaluate the spatial scale of stimulation region. We defined equivalent diameter (ED) to quantitatively present the stimulation region. But ED does not mean the direct activation region of the brain circuits. No published data have specifically shown the activation region stimulated by ultrasound. Furthermore, success rates of 5 MHz and 1 MHz stimuli were calculated to quantify the stimulation regions.

Figure 5b shows that the values of ED5 (ranging from 0.29 ± 0.08 mm to 0.40 ± 0.14 mm) are much smaller than that of ED1 (ranging from 0.83 ± 0.11 mm to 0.91 ± 0.21 mm). Moreover, Fig. 5c shows that the success rate of 5 MHz ultrasound decreased greatly when the stimulation site was moved away from the reference point, but the situation is not the same for 1 MHz ultrasound. It suggests that the effective region of neuro-stimulation by 5 MHz ultrasound is much smaller than that of 1 MHz. In other words, the anatomical specificity of ultrasound stimulation was greatly improved by high frequency ultrasound. We also found that the ED values of 5 MHz and 1 MHz ultrasound were both less than the −3 dB focal widths of 5 MHz and 1 MHz transducers, respectively. This implied that the diameter of the effective stimulation target may be smaller than the −3 dB focal widths. Moreover, there is always a symmetric point which is located at contralateral brain. Repeatable and similar stimulation results can be acquired when stimulating the symmetric points.

Figure 5d illustrates the latency of EMG responses. The difference between latencies of 1 MHz and 5 MHz ultrasound is distinguish (p < 0.001). The mean latency of 5 MHz is less than 100 ms, while that of 1 MHz is longer than 200 ms. These values are different from the result of 22.65 ± 1.70 ms^17^, but are similar with the result of 171 ± 63 ms^31^. The reason why differences existed between 1 MHz and 5 MHz stimulation might relate to the quantity and functions of those neural circuits which were stimulated by ultrasound, and might relate to the temperature elevation.

In this study, 5 MHz ultrasound stimulation would induce temperature elevation of 1.6 °C. According to the former studies^32^,^33^, such temperature elevations are considered to be safe for brain tissue, though may have some influences to the neural activities. If shorten the length of ultrasound waveform, the temperature elevation would greatly decrease accordingly. On the other hand, the results of HE stain illustrate that 5 MHz ultrasound did not induce morphological changes in the stimulated regions. It implies that the proposed 5 MHz ultrasound is safe for neuro-stimulation.

0.5 MHz and 2.25 MHz ultrasound frequencies were also tested for the neuro-stimulation in the preliminary experiments. The results showed that both 0.5 MHz and 2.25 MHz ultrasound also had the ability to evoke motor responses. We found that, the 0.5 MHz and 1 MHz ultrasound behaved similarly in the stimulation effects, since they had close frequencies. For 2.25 MHz ultrasound, it did not possess any obvious advantages compared with 5 MHz ultrasound considering the dimensional scale of mouse skull. Therefore, 5 MHz is proposed in this manuscript. Compared with low frequency ultrasound, higher frequency ultrasound stimulation is more suitable for explore smaller neural circuits which are sensitive to ultrasound, thus become an interesting method for neuroscience research.

## Methods

### Acoustic attenuation and compensation

In order to quantitatively evaluate the influence of mouse skull on the ultrasound energy attenuation with different working frequencies, the acoustic intensity maps were acquired by a 3D ultrasound intensity measurement system (UMS3, Precision acoustics, Dorchester, UK). An ultrasound transducer (V314-1 MHz or V308-5 MHz, 0.75 in., F = 1.5 in., Olympus NDT, Waltham, MA, USA), a needle hydrophone (SN2010, 0.5 mm probe, Precision acoustics, Dorchester, UK), and a mouse skull were sunk into a tank which was filled with degassed water. By scanning the skull with the moving hydrophone, the acoustic intensities after passing through the skull at each scanning point were acquired and plotted in a pseudo-color map with 41*41 points. Similar map without skull was also acquired for comparison. Acoustic compensation was adapted to 5 MHz, as higher frequency suffering higher attenuation loss theoretically. The driven signal was increased proportionally according to the measured results.

### Animal preparation and anesthesia administration

All animal experiments described in this work were approved by the Institutional Ethical Committee of Animal Experimentation of Shenzhen Institutes of Advanced Technology (Chinese Academy of Sciences). The experiments (Certificate number: SIAT-IRB-150203-YGS-QWB-A0088) were carried out strictly in accordance with governmental and international guidelines on animal experimentation. All efforts were made to minimize the usage amount of animals and the suffering during experiments according to the request of Biosafety and Animal Ethics. 10 male C57BL/6 mice, 8–10 weeks old, 21 g (+/−15%) in body weight were used. The mouse was first anesthetized by intraperitoneal injection of ketamine (70 mg/kg) and xylazine (7 mg/kg) cocktail. After that, the fur over mouse’s head was cropped by scissor and removed by depilatory. Then the mouse was laid prone on an automatic heating pad (69002, RWD Co., Shenzhen, China) at 37 degrees centigrade and with its head gently immobilized using stereotaxic frame (68028, RWD Co., Shenzhen, China). Ophthalmic ointment was used to protect its eyes from dry. Acoustic gel was applied and gently kneaded on the scalp. The mouse would get into light anesthesia level gradually with time going on. To maintain the mouse at a stable low level anesthesia stage, microinjection of ketamine (30 mg/kg/h) and sodium chloride solution was applied by intraperitoneal injection using a microinjection pump (0.1 ml/h) and a 30-gauge needle.

### Ultrasound stimulation setup

**Figure 7:**
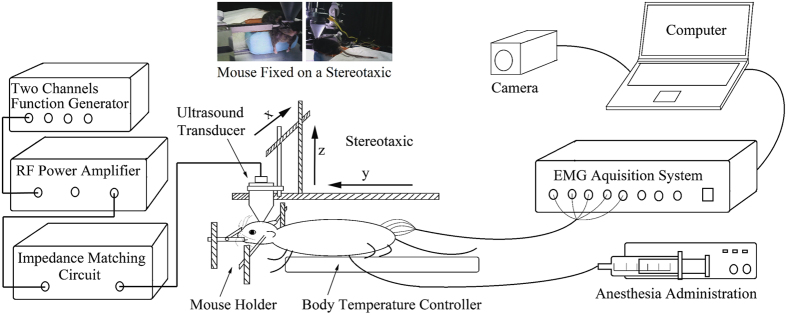
Experimental setup for the ultrasound brain stimulation. The waveforms were generated by a function generator and then amplified by a RF power amplifier. An impedance matching circuit was used to improve the efficiency of the driving circuit. Ultrasound transducer was positioned by a stereotaxic and used to perform brain stimulation at any targets of mouse brain. The mouse was laid on a body temperature controller and fixed by a mouse holder. The motion responses were captured by a camera. The EMG signals were recorded by fine wire stainless steel electrodes and an EMG acquisition system. Micro-injection pump was used for additional anesthesia administration. Photos of a mouse fixed on the stereotaxic were shown on the top.

The schematic of ultrasound stimulation setup is shown in Fig. 7. A function generator (DG5072, Rogol Inc., Beijing, China) with two independent output channels, was used to generate low voltage stimulus sequence. The sequence was then amplified by a RF power amplifier (AR150A100B, AR Europe, Bothell, USA) working at 46 dB gain, and used to drive a transducer to produce ultrasound. An impedance matching circuit was connected between the RF power amplifier and ultrasound transducer to improve the driving efficiency. Ultrasound transducers with identical f-number were used to conduct the ultrasound stimulation. The stimulation site was controlled by a stereotaxic apparatus. The transducer was fixed on a moving bar of the stereotaxic frame and moved above the skull of mouse with 0.01 mm steps in a 3D manner. Stimulation effect was evaluated by EMG signals recorded by an EMG acquisition system (MedLab-U8C502, MedEase Ltd., Nanjing, China). The motion responses of mouse were captured by a camera (HD1080P, Aoni Ltd., Shenzhen, China) in real-time. The EMG data and videos were stored in a computer for offline processing.

### Acquisition and process of EMG signals

The EMG signals were collected from tail muscles using fine-wire stainless steel electrodes^14^. The signals were amplified, filtered and recorded by the EMG acquisition system. The main parameters for acquisition setting were X1000 gain, 0.3 Hz to 1000 Hz band-pass filter, no notch filter, and 5 kHz sampling frequency. The EMG data were then processed by Matlab (MathWorks, Natick, MA, USA). They were first filtered with 50 Hz notch filter and 20 Hz high-pass filter and then rectified and sectioned into final data sets according to the stimulus synchronous signal. The onset and offset time points of each final data set were chosen according to a threshold value of triple standard deviations of the background noises^23^. The peak EMG amplitude was defined as the maximum value within each final data set. The time between stimulus onset and the active reaction was defined as response latency. Success rate was the percentage of the number of valid stimuli divided by the total number of stimuli. A valid stimulation was defined when its peak EMG amplitude was three times higher than standard deviations of the background noises.

### Selection of stimulation locations

**Figure 8:**
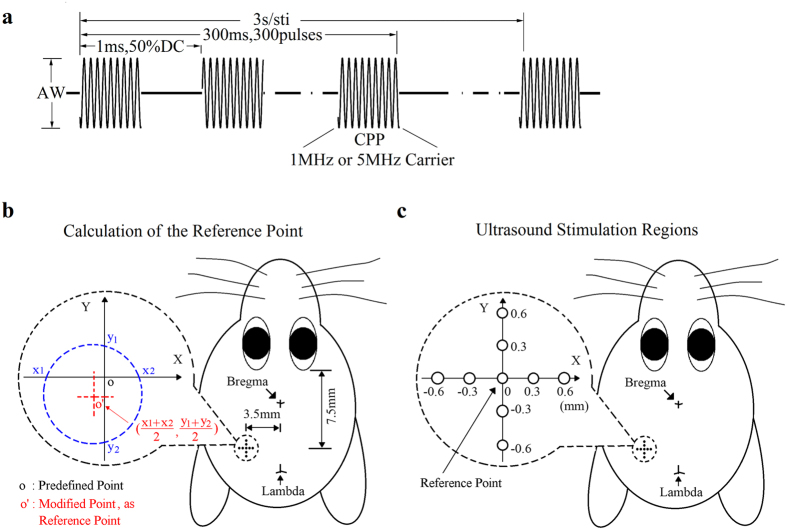
Schematic diagram of ultrasound stimulus waveform and locations. (**a**) A waveform used for the stimulation. (**b**) Illustration of finding the reference point for ultrasound stimulation. The predefined point o is at 3.5 mm lateral to midline and 7.5 mm posterior to the rear corner of mouse eyes. The bregma is not used as it is still covered by the scalp. The x1, x2, y1, and y2 locations are the critical points, where EMG signals just appear while moving the stimulation transducer along X axis and Y axis. The modified (reference) point o’ is finally located at the center of the four critical points. (**c**) Illustration of ultrasound stimulation site on a mouse. The nine circles represent stimulation sites, which are 0.3 mm apart from each other, and arranged on X and Y axis. The central circle represents the reference point, which is found according to (**b**).

The ultrasound stimulation sequence was similar with the published work^15^ (Fig. 8a). By scanning the ultrasound transducer on the mouse skull by step of 1 mm along X axis and Y axis, motion response were evaluated by EMG amplitude at each stimulation site. Such preliminary experiments shown that not all of the positions on the mouse skull could be used to evoke motion response. One of the most sensitive stimulation sites locates around 3.5 mm lateral to the midline and 7.5 mm posterior to the rear corner of mouse eyes. This sensitive site is named as predefined point, more accurate position for each individual mouse can be explored by finding the critical points (Fig. 8b). The x1, x2, y1, and y2 locations are the critical points, where EMG signals just appear while moving the stimulation transducer along X axis and Y axis. The modified (reference) point o’ is finally located at the center of the four critical points, and is used for the following stimulation experiment for each mouse.

### Stimulation with high frequency ultrasound

In order to evaluate which acoustic intensity is the best for the neuro-stimulation, six I_spta_ values ranging from 130 mW/cm^2^ to 230 mW/cm^2^ were used. The stimulation duration was one minute each time. Furthermore, in order to estimate the stability of stimulation, responses evoked by the six various intensities at three different time frames, i.e. 50 min, 80 min, and 110 min after the first anesthesia injection were also analyzed.

### Comparison between different ultrasound frequencies

In order to compare the neuro-stimulation effect of 5 MHz ultrasound with 1 MHz, the stimulation energy was set in an identical level after compensation. The waveform illustrated in (Fig. 8a) was used to drive the 1 MHz or 5 MHz transducer. Except for two parameters, i.e. the cycles per pulse (CPP) and the amplitude of waveform (AW), all other parameters were kept consistent (50% duty cycle of pulse, 1 ms pulse period, 1 kHz pulse repetition frequency, 300 pulses per stimulus, and 3 second interval). For 1 MHz and 5 MHz ultrasound, CPPs were set to 500 and 2500, respectively, to satisfy the demand of 50% duty cycle, i.e. 0.5 ms pulse duration. The AWs were adjusted to maintain identical ultrasound intensity level of 1 MHz and 5 MHz after passing through the skull.

### Evaluation of the anatomical specificity with different ultrasound frequencies

The focal region of 5 MHz ultrasound is smaller than that of 1 MHz ultrasound theoretically. If the active region in brain circuits is only around the focal region of ultrasound, the specificity of neural activation can be improved by the 5 MHz ultrasound. Figure 8c shows the method to evaluate the stimulation performance with different ultrasound settings. Nine stimulation sites which were 0.3 mm apart from each other were arranged horizontally on X axis and vertically on Y axis. The position of the center stimulation site located at the reference point mentioned before. 1 MHz and 5 MHz transducer were used to perform stimulation according to the allocated positions. The stimulation duration was one minute for each point. The successive EMG signals from each duration period were recorded for offline process.

A quantification parameter named equivalent diameter (ED) was used to characterize the anatomical stimulation specificity of 1 MHz and 5 MHz ultrasound. It is the distance between two points which are located in a fitted curve and having their normalized peak EMG amplitude value with 0.707. ED1 and ED5 are the ED value for 1 MHz and 5 MHz ultrasound along the Y axis respectively. Statistics analysis of ED values from five mice between X and Y axis was also achieved.

The success rate at three stimulation sites, which located at 0 mm, 0.3 mm, and 0.6 mm from the reference point, were also noted and evaluated for the comparison. The response latencies at 0 mm, and 0.3 mm sites were also under statistics analysis.

### The evaluation of safety with temperature measurement and hematoxylin-eosin (HE) stain

In order to evaluate the safety issue of using 5 MHz ultrasound for neuro-stimulation, the elevated temperature was recorded and HE stains were also applied. An optical fiber based thermal sensor (Fots-Dina-1000-s, Indigo Precision Technologies Co. Ltd, Beijing, China) was inserted into the brain tissue to measure the temperature of the stimulating points. The peak value of temperature elevation was recorded during the stimulation period. In terms of HE stain process, 10 mice were used for safety test, i.e. 4 mice for 1 MHz stimuli, 4 mice for 5 MHz stimuli and 2 mice for control group without any ultrasound stimuli. The stimulus power and other parameters were consistent with those used on the normal stimulation study, and the stimulation duration was 30 min. Mice were deeply anesthetized with pentobarbital sodium, followed by perfusion of 4% paraformaldehyde for 10 min at 4 °C. The brains were removed and stored in the 4% paraformaldehyde solution overnight at 4 °C. The samples were dehydrated in a graded ethanol series, and then embedded in paraffin^34^. Coronal sections were cut at 5μm in thickness using rotary microtome (RM2235, Leica Biosystems Nussloch GmbH, Heidelberger, Germany) and standard HE stain was performed^35^. Brain slices were observed on microscope (Eclipse Ni-U, Nikon Instruments Inc., Tokyo, Japan) and captured by camera (Ds-Ri1, Nikon Instruments Inc., Tokyo, Japan).

## Additional Information

**How to cite this article**: Li, G.-F. *et al.* Improved Anatomical Specificity of Non-invasive Neuro-stimulation by High Frequency (5 MHz) Ultrasound. *Sci. Rep.* **6**, 24738; doi: 10.1038/srep24738 (2016).

## Supplementary Material

Supplementary Video 1

Supplementary Information
