# Supplementary material for: Improved Anatomical Specificity of Non-invasive Neuro-stimulation by High Frequency (5 MHz) Ultrasound
Source: Sci Rep. 2016 Apr 20;6:24738. doi: 10.1038/srep24738 (PMC4837374; doi:10.1038/srep24738)
Supplement: Supplementary Information [file srep24738-s2.doc]

**Improved Anatomical Specificity of Non-invasive Neuro-stimulation by High Frequency (5 MHz) Ultrasound**

## Guo-Feng Li^1,2^, Hui-Xia Zhao^1^, Hui Zhou^1^, Fei Yan^1^, Jing-Yao Wang^1^, Chang-Xi Xu^1^, Cong-Zhi Wang^1^, Li-Li Niu^1^, Long Meng^1^, Song Wu^3^, Huai-Ling Zhang^2^, Wei-Bao Qiu^1^*, & Hai-Rong Zheng^1^*

^1^ Institute of Biomedical and Health Engineering, Shenzhen Institutes of Advanced Technology, Chinese Academy of Sciences, Shenzhen, 518055, China, ^2^ School of Information Engineering, Guangdong Medical University, Dongguan, 523808, China, ^3^ Shenzhen Luohu People's Hospital, Shenzhen, 518001, China

* **Co-corresponding authors:**

Wei-Bao Qiu, Ph.D.

Address: 1068 Xueyuan Avenue, Shenzhen University Town, Shenzhen, 518055, China

Tel: +86-755-86392284;

E-mail: [wb.qiu@siat.ac.cn](mailto:wb.qiu@siat.ac.cn)

Hai-Rong Zheng, Ph.D.

Address: 1068 Xueyuan Avenue, Shenzhen University Town, Shenzhen, 518055, China

Tel: +86-755-86392244; Fax: +86-755-86392299;

E-mail: [hr.zheng@siat.ac.cn](mailto:hr.zheng@siat.ac.cn)

**Video Legends:**

The supplementary video illustrates motor responses evoked by transcranial ultrasound stimulation on mouse brain. The stimulus parameters include 5MHz ultrasound frequency, 50% duty cycle of pulse, 1kHz pulse repetition frequency, 300ms stimulation duration, 3s stimulation period, and I_spta_ of 210 mW/cm^2^. The left part of the frame shows the EMG waveforms collected from muscles of right forelimb (red) and tail (blue), with synchronous waveform (green) indicating ultrasound stimulation phase. The right part of the frame shows the motor responses of a mouse under the brain stimulation of 5MHz ultrasound. The red flash indicated the phase of stimulation.
